# Validation of a targeted gene panel sequencing for the diagnosis of hereditary chronic liver diseases

**DOI:** 10.3389/fgene.2023.1137016

**Published:** 2023-06-14

**Authors:** Luisa Ronzoni, Ilaria Marini, Giulia Passignani, Francesco Malvestiti, Daniele Marchelli, Cristiana Bianco, Serena Pelusi, Daniele Prati, Luca Valenti

**Affiliations:** ^1^ Biological Resource Centre, Precision Medicine Lab, Transfusion Medicine, Fondazione IRCCS Ca’ Granda Ospedale Maggiore Policlinico Milano, Milano, Italy; ^2^Department of Pathophysiology and Transplantation, Università Degli Studi di Milano, Milano, Italy

**Keywords:** hereditary chronic liver diseases, targeted sequencing, gene panel design, whole exome sequencing (WES), next-generation sequencing, NGS

## Abstract

**Background:** The cause of chronic liver diseases (CLD) remains undiagnosed in up to 30% of adult patients. Whole-Exome Sequencing (WES) can improve the diagnostic rate of genetic conditions, but it is not yet widely available, due to the costs and the difficulties in results interpretation. Targeted panel sequencing (TS) represents an alternative more focused diagnostic approach.

**Aims:** To validate a customized TS for hereditary CLD diagnosis.

**Methods:** We designed a customized panel including 82 CLD-associated genes (iron overload, lipid metabolism, cholestatic diseases, storage diseases, specific hereditary CLD and susceptibility to liver diseases). DNA samples from 19 unrelated adult patients with undiagnosed CLD were analyzed by both TS (HaloPlex) and WES (SureSelect Human All Exon kit v5) and the diagnostic performances were compared.

**Results:** The mean depth of coverage of TS-targeted regions was higher with TS than WES (300x vs. 102x; *p* < 0.0001). Moreover, TS yielded a higher average coverage per gene and lower fraction of exons with low coverage (*p* < 0.0001). Overall, 374 unique variants were identified across all samples, 98 of which were classified as “Pathogenic” or “Likely Pathogenic” with a high functional impact (HFI). The majority of HFI variants (91%) were detected by both methods; 6 were uniquely identified by TS and 3 by WES. Discrepancies in variant calling were mainly due to variability in read depth and insufficient coverage in the corresponding target regions. All variants were confirmed by Sanger sequencing except two uniquely detected by TS. Detection rate and specificity for variants in TS-targeted regions of TS were 96.9% and 97.9% respectively, whereas those of WES were 95.8% and 100%, respectively.

**Conclusion:** TS was confirmed to be a valid first-tier genetic test, with an average mean depth per gene higher than WES and a comparable detection rate and specificity.

## Introduction

Chronic liver disease (CLD) is a major health concern whose prevalence and related morbidity and mortality continues to rise, accounting for over 2 million deaths annually worldwide (Karlsen et al., 2022). Despite advances in clinical and instrumental workup, the causes of CLD remain unknown in a large fraction of adults, ranging from 14% to 30% (Vilarinho and Mistry, 2019; Gao et al., 2021). In the last decade, advances in genetic technologies, using a next-generation sequencing (NGS) approach, have enhanced and broadened the comprehension of the causes of rare genetic disorders, increasing the understanding of CLD etiology and allowing to diagnose a large fraction of cases previously classified as “cryptogenic.” Whole-exome sequencing (WES) has been shown to yield a high diagnostic rate not only in pediatric population but also in adults with undiagnosed CLD (Pelusi et al., 2022; Valenti and Ronzoni, 2022; Zheng et al., 2022). Although WES is already available in clinical practice for some specific indications, there are some remaining challenges on the road to a more extensive implementation. These include the need of specific expertise related to both data analysis and interpretation, and the time and costs associated with analysis and diagnostic interpretation of genomic data. Furthermore, WES can detect incidental actionable inherited conditions, not related to primary indications for testing, which raises ethical issues (Hakim et al., 2019; Valenti et al., 2019).

Customized targeted gene panel sequencing (TS) focused on a limited set of genes specific for CLD is an alternative to WES. TS has the advantage of providing higher coverage that increases analytical sensitivity even in the detection of mosaicism, of being easier to interpret and of avoiding incidental secondary findings.

In this study, we optimized a customized TS for hereditary CLD diagnosis and compared its performance to that of WES in a cohort of 19 unrelated adult patients with undiagnosed CLD.

## Materials and methods

### Patients’ selection and study design

From January 2021 to December 2021, 19 unrelated adult patients with undiagnosed CLD with a suspected hereditary etiology, referred to the Hepatology outpatient service and Precision Medicine Lab of the Department of Pathophysiology and Transplantation, Transfusion Medicine Unit of the Fondazione IRCCS Ca’ Granda Ospedale Maggiore Policlinico of Milan, for clinical and genetic evaluation, were included into the study. All were of European ancestry. Viral and autoimmune hepatitis were ruled out using standard clinical and laboratory evaluation; CLD due to alcohol abuse were excluded.

For each patient, a peripheral blood DNA sample was analyzed by both TS and WES, filtered for the genes included in target panel design, and their performances were compared.

The study protocol was approved by the Ethical Committee of Fondazione IRCCS Ca’ Granda (CE 125_2018bis). Written informed consent for genetic analysis was obtained from each patient.

### Custom targeted gene panel design

Based on data from the Human Genome Mutation Database (HGMD), Online Mendelian Inheritance in Man (OMIM) database (http://www.ncbi.nlm.nih.gov/omim), and an extensive literature review using PubMed, we selected 82 disease-causing genes associated to CLD, classified into six categories: iron overload, lipid metabolism, cholestatic diseases, storage diseases, specific hereditary CLD and genes associated to susceptibility to liver diseases (Supplementary Table S1). For each gene, we considered the coding exons with 25 flanking bp; selected 5'-/3′-UTRs and promoter regions known to harbor pathogenic variants were also included. Probes specific for the selected regions were designed with the HaloPlex online design tool (SureDesign, Agilent Technologies Inc.), using GRCh37-hg19 as reference genome.

### DNA extraction

DNA was extracted from peripheral blood and quantified by a Qubit 2.0 analyzer using the Qubit dsDNA BR Assay Kit (Thermo-Fisher, Waltham, MA, United States). Sample purity was evaluated using a Nanodrop 1,000 spectrophotometer (Thermo-Fisher, Waltham, MA, United States) and integrity was assessed by gel electrophoresis and using the Agilent 2,200 TapeStation System and the Genomic DNA ScreenTape assay for DNA Integrity Number (DIN) assessment (Agilent Technologies, Santa Clara, CA, United States). Samples with a 260/280 ratio of absorbance ≥1.8 and a DIN ≥6.4 were used.

### Targeted sequencing and analysis

Amplicon libraries were prepared from genomic DNA using the HaloPlex Target Enrichment System specific for the designed CLD panel (Agilent, Cernusco sul Naviglio, Milan, Italy), according to the manufacture’s protocol. Sequencing was performed on MiSeq platform (Illumina, San Diego, CA). FASTQ files were analyzed using SureCall version 4.2.2 (Agilent, Cernusco sul Naviglio, Milan, Italy). Briefly, adapter sequences and lower-quality bases were removed; reads were aligned to reference genome (GRCh37-hg19) using Burrows Wheeler Aligner (BWA)-MEM algorithm. Variant calling was performed using the algorithm SNPPET SNP of SureCall. A coverage depth cutoff of 10x was applied. The obtained Variant Call Format (vcf) files were analyzed and annotated in wANNOVAR server (http://wannovar.usc.edu).

### Whole exome sequencing and analysis

Genomic DNA libraries were enriched for WES by the SureSelect Human All Exon v5 kit (Agilent, Cernusco sul Naviglio, Milan, Italy). Sequencing was performed on the NextSeq 550 platform (Illumina, San Diego, CA). Variants calling and analysis were performed using a validate pipeline previously described (Pelusi et al., 2019; Baselli et al., 2022).

Briefly, raw reads quality control was performed using FastQC software (Brabaham bioinformatics, Cambridge, United Kingdom). Reads mapping on human GRCh37 genome was performed using MEM algorithm of Burrows Wheeler Aligner (BWA) version 0.7.10. Reads with low quality alignments and duplicate reads have been filtered out using SAMtools to generate high quality bam (HQ-BAM) files and mapping quality control was performed using Picard-tools and Bedtools softwares. Variants calling was performed following Genome Analysis Toolkit (GATK) best practices. Variants quality score log-odds (VQSLOD) above 99% tranche were considered true positives and variants present in <20% of total reads discarded; indel left-normalization was performed using BCFtools software and variants annotation using both variant effect predictor (VEP) and ANNOVAR tools. Only the variants included in the regions covered by TS were considered for the analysis. A coverage depth cutoff of 10x was applied. Pathogenic variants analysis was performed using ClinVar database. Variants pathogenicity was predicted using public algorithms such as CADD (Combined Annotation Dependent Depletion) and a score >20 was selected in order to classify the variants with a potential high functional impact (HFI) (Baselli et al., 2022).

### Sanger sequencing

Selected variants, uniquely detected by TS or WES, were confirmed by Sanger sequencing. The specific PCR primers were designed using both Primer Design Tool-NCBI (https://www.ncbi.nlm.nih.gov/tools/primer-blast/) and Primer3 software (https://primer3.ut.ee/); primers sequences are available upon request. Amplicons were sequenced using the Big Terminator v3.1 cycle sequencing kit (Applied Biosystems™, Thermo-Fisher, Waltham, MA, United States) and a Sanger Sequencing 3,500 Dx Series Genetic Analyzer (Applied Biosystems™, Thermo-Fisher, Waltham, MA, United States). Sequences were first analyzed using the Sequencing Analysis Software v7.0 (Applied Biosystems™, Thermo-Fisher, Waltham, MA, United States) and then compared to reference genome using BLAST software (https://blast.ncbi.nlm.nih.gov/Blast.cgi).

### Statistical analysis

For statistical analysis, non-parametric Wilcoxon Signed Ranked test or Chi-squared test were used, as appropriate. *p* values < 0.05 (two tailed) were considered statistically significant.

## Results

### TS and WES coverage

The mean depth of coverage of the TS-targeted regions obtained with TS was higher, although more variable, than that obtained with WES in all the samples, with an average depth of 300x for TS (range 122-738x) and 102x for WES (range 94-119x), and with 97% and 87% of the targeted regions covered at 10x sequence depth in TS and WES respectively (Table 1).

**TABLE 1:** Mean depth of coverage and percentage of targeted regions with coverage >10x in TS and WES.

Sample	TS mean depth of coverage	% Targeted regions with coverage >10x in TS	WES mean depth of coverage in TS regions	% Targeted regions with coverage >10x in WES
1	206	97	95	85
2	601	99	102	85
3	738	99	96	85
4	475	99	96	86
5	455	97	94	85
6	189	99	103	85
7	269	97	102	87
8	212	98	96	87
9	253	98	96	87
10	197	98	102	90
11	193	97	100	90
12	179	97	106	91
13	178	94	94	89
14	122	93	103	91
15	135	91	97	86
16	658	99	119	92
17	229	98	112	87
18	200	98	115	88
19	221	98	105	87
mean	301	97	102	88
sd	187	2	7	2

**FIGURE 1:**
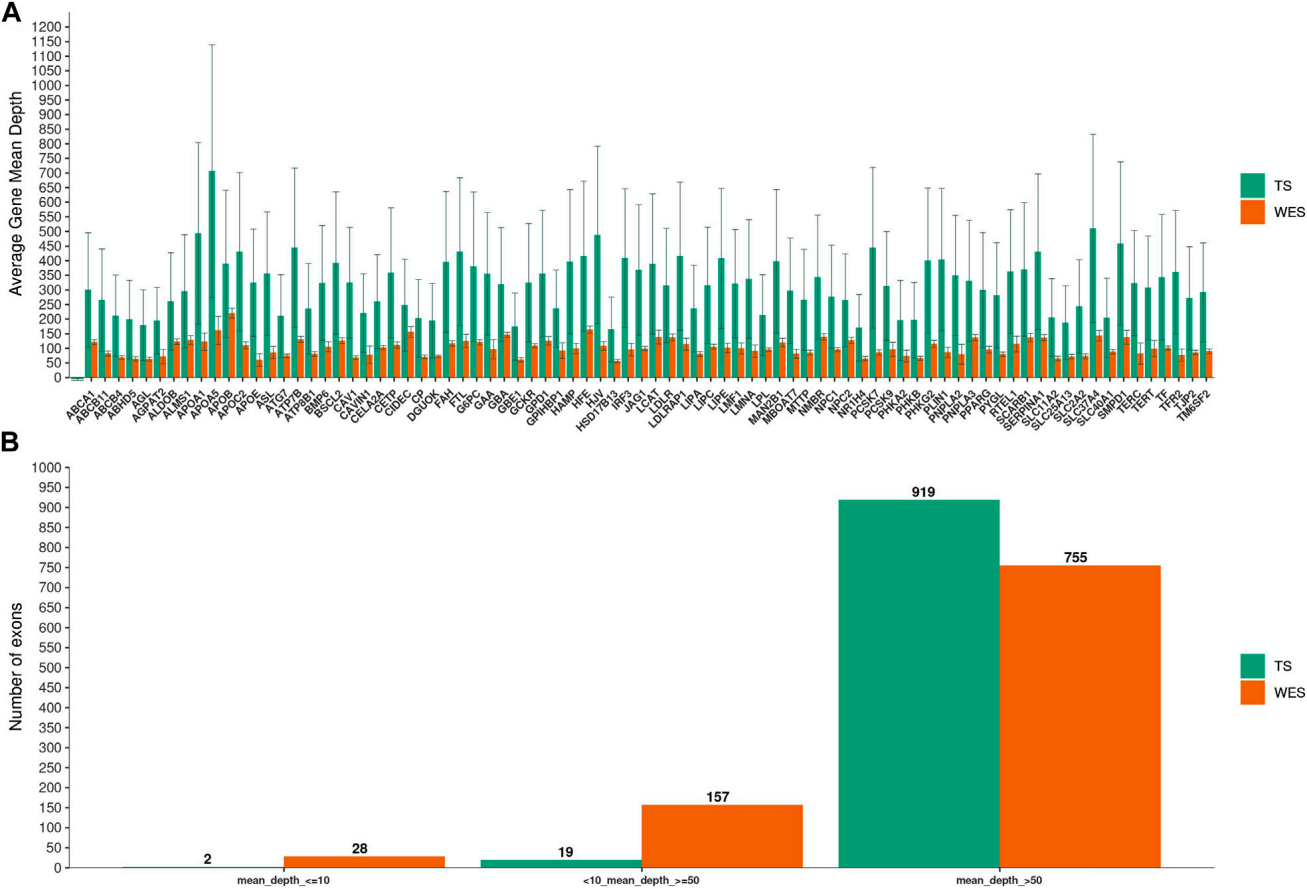
Mean depth of coverage per chronic liver diseases genes. In **(A)**, Targeted Sequencing (TS) yielded significantly higher average mean depth per gene compared to Whole-Exome Sequencing (WES). Bars indicate standard deviation. In **(B)**, the fraction of exons covered to the given depth for each methods is shown.

We next examined the coverage on a gene-by-gene basis by pooling data from all samples. TS yielded higher average mean depth per gene than WES (*p* = 3.7e-15) (Figure 1A), and had substantially fewer exons with less than tenfold mean depth (0.2% vs 2.8%; *p* = 2.1e-06) (Figure 1B)

### Variants detection

In order to compare TS and WES performance in variants detection, we first considered the total number of variants identified across all samples in the regions covered by TS design (exonic, UTR and splice-site variants).

Overall, 374 unique variants were identified across all samples, 288 of which (77%) were identified by both methods, 66 (18%) uniquely by TS and 20 (5%) by WES only. We then restricted the analysis to variants with a potential HFI. Specifically, we considered exonic non-synonymous (missense, frameshift, nonsense), UTR or splice-site variants. Among these, we next selected variants with at least one of the following characteristics: a) reported as “Pathogenic” or “Likely Pathogenic” in the ClinVar database; b) rare variants of unknown significance (VUS) with a minor allele frequency (MAF) < 0.005 according to public database (ExAC_NFE); or c) VUS with a MAF between 0.005 and 0.05 and a CADD score >20.

We identified a total of 98 HFI variants, 89 of which were detected by both methods. We next focused on the 9 HFI variants uniquely detected by TS or WES to analyze the possible reasons underlying these discrepancies. All these variants were also analyzed by Sanger sequencing.

(i) Variants uniquely detected by TS

All the six variants uniquely detected by TS were exonic non-synonymous ones (Table 2). Two variants (rs201365106 in *RTEL1* and rs78250081 in *SMPD1* gene) were not confirmed by Sanger sequencing. One variant (rs201365106) was a Single Nucleotide Variant (SNV) in a GC-rich region of *RTEL1* gene, while the other one (rs78250081) was a SNV affecting a repetitive hexanucleotide GCTGGC sequence in a polymorphic region of *SMPD1* gene (Supplementary Figure S1). In both cases, the mis-alignment of some TS sequencing reads led to incorrect base identification with a false positive variant calling. The other four variants uniquely detected by TS were confirmed by Sanger sequencing. The causes for missing call by WES for these variants were different. In two cases, both involving the *PCSK7* gene, the corresponding regions in WES had insufficient mean depth (>10x) to make an accurate base call (Supplementary Figures S2A, S2B); this could be related to the WES capture design that did not contain enough baits for these *PCSK7* regions. In the other two cases, analysis of WES raw data showed that discrepancies were due to low SNV quality score estimated by the SAMtools variant identification software.

(ii) Variants uniquely detected by WES

**TABLE 2:** Variants with high functional impact identified by TS only.

Gene	Variant ID	Nucleotide variant	Consequence	ClinVar_SIG	CADD score	ExAC_NFE	Sanger confirmation	% GC-content in variant region	Sequencing depth of variant region in TS	Sequencing depth of corresponding region in WES	Proposed reason for discrepancies
*PCSK7*	rs781628227	c.2347C>G	Exonic non-synonymous	Not_Found	24.5	0	Confirmed	55.9	569x	0	Insufficient coverage in WES
*CIDEC*	rs757906759	c.209C>T	Exonic non-synonymous	Not_Found	32	0.00004496	Confirmed	51.3	436x	298x	No VQSR filtering pass in WES pipeline
*SMPD1*	rs78250081	c.113C>T	Exonic non-synonymous	Not_Found	17.7	0.00005943	Not Confirmed	73.5	610x	54x	False-positive variant calling in TS due to mis-alignment in repetitive region
*CP*	rs61733458	c.1652C>T	Exonic non-synonymous	Benign	29.7	0.0301	Confirmed	44.8	199x	94x	No VQSR filtering pass in WES pipeline
*RTEL1*	rs201365106	c.232G>A	Exonic non-synonymous	Uncertain significance	2.8	0	Not Confirmed	75	498x	275x	False-positive variant calling in TS due to mis-alignment in CG-rich region
*PCSK7*	------	c.1737C>A	Exonic non-synonymous	Not_Found	41	0	Confirmed	60	754x	0	Insufficient coverage in WES

All the three variants uniquely detected by WES were exonic and were confirmed by Sanger sequencing (Table 3). The main cause of missing call by TS was the low mean depth of the corresponding region in Target Panel design.

**TABLE 3:** Variants with high functional impact identified by WES only.

Gene	Variant ID	Nucleotide variant	Consequence	ClinVar_SIG	CADD score	ExAC_NFE	Sanger confirmation	% GC-content in variant region	Sequencing depth of variant region in WES	Sequencing depth of corresponding region in TS	Proposed reason for discrepancies
*CAV1*	rs150368249	c.463G>A	Exonic non-synonymous	Likely benign	16.3	0.0002472	Confirmed	46.8	165x	15x	Insufficient coverage in TS
*PHKA2*	rs145406549	c.785T>G	Exonic non-synonymous	Not_Found	27.9	0.0003297	Confirmed	40.2	219x	14x	Insufficient coverage in TS
*PHKB*	rs151155518	c.500A>G	Exonic non-synonymous	Conflicting interpretations of pathogenicity	27.2	0.006614	Confirmed	44.4	74x	10x	Insufficient coverage in TS

**FIGURE 2:**
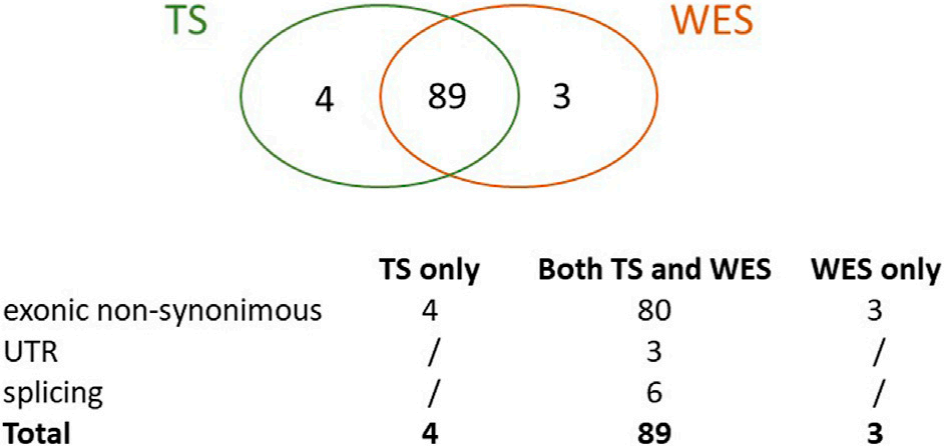
True variants with high functional impact detected by TS, WES or both. The Venn graph and the type of variants detected are shown.

Overall, we identified a total of 96 true HFI variants, 89 of which (93%) were detected by both methods, 4 (4%) were uniquely identified by TS and 3 (3%) by WES only (Figure 2).

### TS and WES detection rate

TS and WES detection rate was calculated as the ability of TS or WES to identify a SNV over the total of detected variants. Variants detected by either one of the two methods and confirmed by Sanger sequencing were considered true positive. Detection rate of TS was 96.9% while that of WES was 95.8%. TS specificity was 97.9% and that of WES was 100%.

## Discussion

TS customized for specific subsets of candidate genes has been proposed as a first-tier diagnostic approach for several hereditary diseases (Consugar et al., 2015; Butz et al., 2021; Oh et al., 2021). Compared to WES, TS has the advantage to be more focused on disease-causing genes, allowing a higher depth of coverage in targeted regions and producing a lower volume of data, thereby reducing costs and turnaround time. However, the majority of the studies supporting the higher diagnostic yields of TS did not include any cross comparisons with the diagnostic yield of WES for the same disease, or TS performance was compared to that of WES performed on different samples, or only theoretically estimated by bioinformatics approaches (LaDuca et al., 2017).

In this study, we designed a customized TS panel including 82 liver-related genes, selected on the basis of patient phenotypes and through an extensive literature review. Both targeted panel and exome sequencing were successfully performed concurrently on 19 consecutive patients.

We confirmed that TS had a higher mean depth of coverage compared to WES in the targeted regions. Particularly, TS had an average mean depth per gene significantly higher than WES and substantially fewer exons with less than tenfold mean depth (*p*-value<0.0001), allowing a deeper evaluation of specific target genes and regions.

Although the majority of variants with a functional or likely functional impact (89 over 98: 91%) were recognized by both methods, some variants (9 over 98) were identified by TS only (6) or WES only (3). The main reason of these discrepancies, accounting for 5 inconsistencies over a total of 9 (55%), was related to the insufficient read depth leading to missing variants call (2 missed by WES and 3 by TS). Two variants recognized by TS only were false positives, not confirmed by Sanger sequencing. These false positive calls were most likely due to alignment ambiguity in repetitive or CG-rich regions, leading to incorrect base identification by the software used for the analysis. Two other variants identified by TS only and confirmed by Sanger sequencing were missed by WES due to the analytical pipeline used for variant calling and filtering. Overall, 4 over 9 discrepancies in variant calling (45%) were accounted for by the analytical pathway rather than by the NGS approach.

Some limitations of our study should be noted. First, NGS analyses (TS and WES) were performed once for each sample, so that intra-sample reproducibility of the results could not be evaluated. This did not allow to quantify the possible role of technical variability, mainly in libraries preparation and capturing steps, that may account for insufficient coverage of specific exons in individual samples and consequently for missing variant calling. However, the strength of the study was that both TS and WES were performed on each sample, allowing a direct comparison between the two methods.

Overall, we confirmed the possible advantage of TS over WES in identifying high functional impact variants in specific cases. In fact, although in the present limited cohort of patients analyzed the detection rate and specificity between the two NGS approach were not significantly different, TS was a less laborious, less expensive and with a reduced turnaround time method. Moreover, based that the clinical utility of NGS methods mainly relied on the ability to accurately isolate or amplify the genes of interest, TS allowed a deeper evaluation of the regions of interest. Despite this advantage, one key drawback of TS was that they may become outdated rather quickly as new disease-related genes are discovered and they did not allow to identify variants in genes or regions not comprised in panel design. As a result, extending to WES as a second-tier genetic test in cases remained undiagnosed after TS, using a hierarchical strategy, would greatly increase the costs of the overall diagnostic workflow. On the other hand, WES had the important advantage of providing evidence about the putative variants related to the disease without previous biased knowledge. This feature can be useful in heterogeneous conditions such as CLD, characterized by variable expressivity and incomplete penetrance.

In conclusion, when the genetic background is well-defined and a specific hereditary CLD is suspected, TS can be used as a first-tier genetic test, supporting clinical and instrumental evaluation. However, when no suspect gene stands behind the clinical phenotype, as in cryptogenic CLD, WES can provide a wider screening option, especially in association with familial segregation studies.

## Data Availability

The genetic data has been summitted to https://www.ebi.ac.uk/eva/. Project: PRJEB62846. Analyses: ERZ18542841.

## Abbreviations

CLD, Chronic Liver Disease; NGS, Next-Generation Sequencing; WES, Whole-exome sequencing; TS, Targeted gene panel Sequencing; HGMD, Human Genome Mutation Database; OMIM, Online Mendelian Inheritance in Man; CADD, Combined Annotation Dependent Depletion; HFI, High Functional Impact; VUS, Variants of Unknown Significance; MAF, Minor Allele Frequency; SNV, Single Nucleotide Variant.
