# Supplementary figures and images for: Validation of a targeted gene panel sequencing for the diagnosis of hereditary chronic liver diseases
Source: Front Genet. 2023 Jun 14;14:1137016. doi: 10.3389/fgene.2023.1137016 (PMC10300275; doi:10.3389/fgene.2023.1137016)

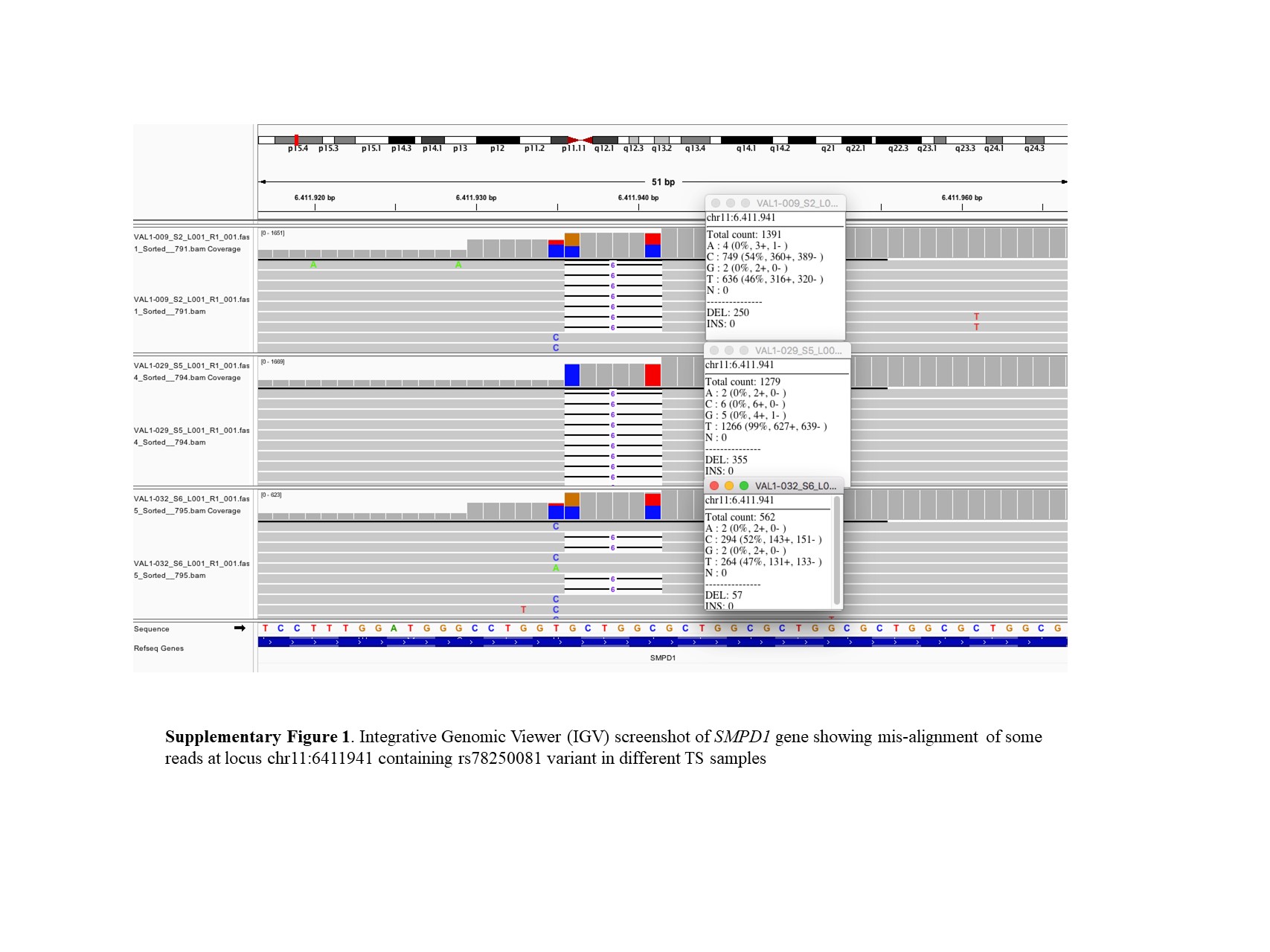

Supplement: Supplementary file 1 [file Image1.JPEG]

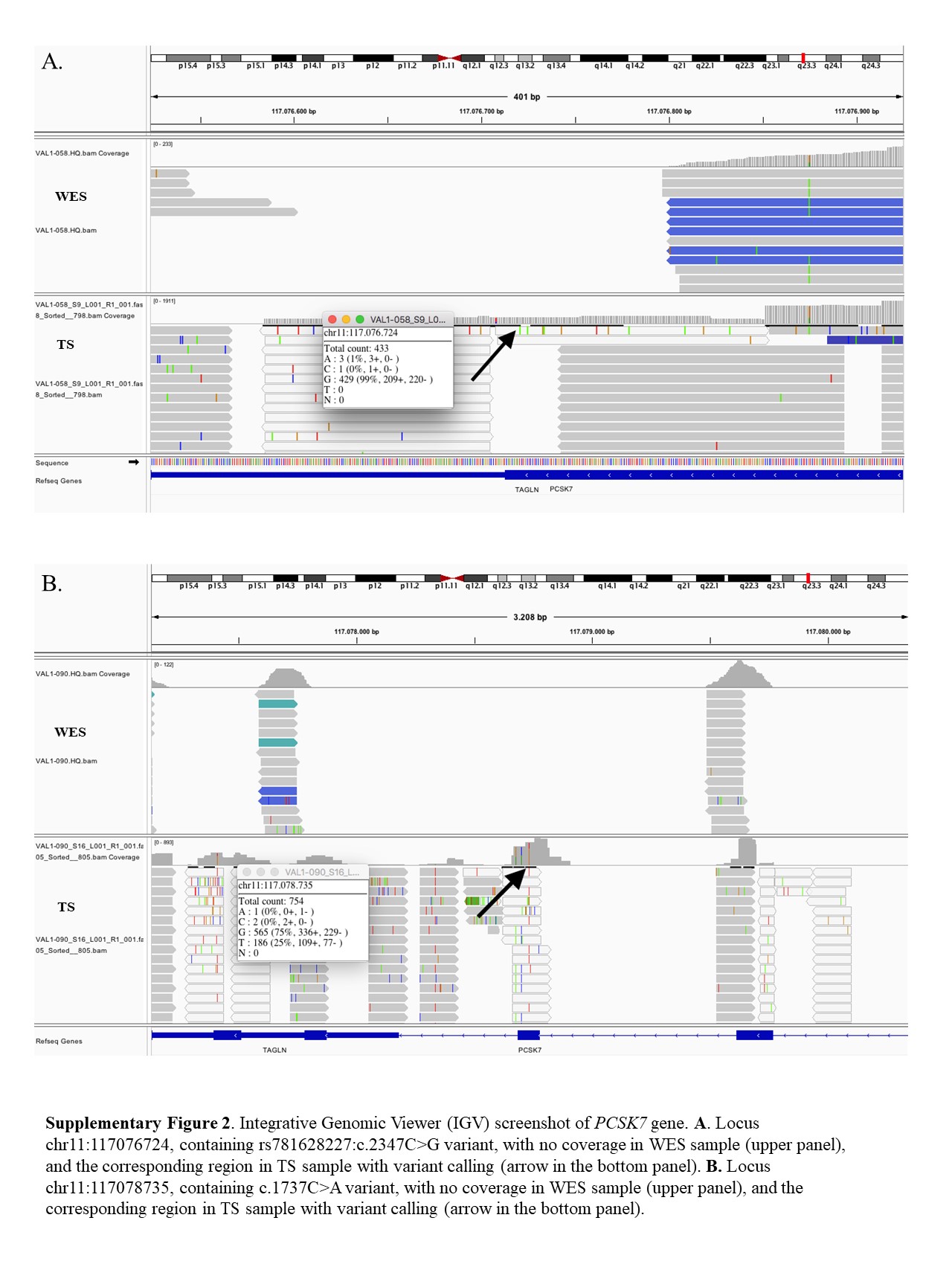

Supplement: Supplementary file 2 [file Image2.JPEG]
