# Supplementary material for: Validation of a targeted gene panel sequencing for the diagnosis of hereditary chronic liver diseases
Source: Front Genet. 2023 Jun 14;14:1137016. doi: 10.3389/fgene.2023.1137016 (PMC10300275; doi:10.3389/fgene.2023.1137016)
Supplement: Supplementary file 3 [file DataSheet1.pdf]

Gene	Cytogenetic location	Gene Accession Number	Inheritance	Associated Disease
**Iron overload**				
HFE	6p22.2	ENSG00000010704	AR	Hemochromatosis, type 1 [1]
HJV	1q21.1	ENSG00000168509	AR	Hemochromatosis, type 2A [1]
HAMP	19q13.12	ENSG00000105697	AR	Hemochromatosis, type 2B [1]
TFR2	7q22.1	ENSG00000106327	AR	Hemochromatosis, type 3 [1]
SLC40A1 *	2q32.2	ENSG00000138449	AD	Hemochromatosis, type 4 [1]
TF	3q22.1	ENSG00000091513	AR	Atransferrinemia [1]
FTL *	19q13.33	ENSG00000087086	AD	Hyperferritinemia-cataract syndrome [2]
SLC11A2	12q13.12	ENSG00000110911	AR	Anemia, hypochromic microcytic, with iron overload 1 [3]
CP	3q24-q25	ENSG00000047457	AR	Hypo/Aceruloplasminemia [4]
BMP6		ENSG00000153162		Hemochromatosis [5]
**Lipid metabolism**				
APOB	2p24.1	ENSG00000084674	AR	Hypobetalipoproteinemia [6]
			AD	Hypercholesterolemia, familial, 2 [7]
LDLR *	19p13.2	ENSG00000130164	AD, AR	Hypercholesterolemia, familial, 1 [7]
LDLRAP1	1p36.11	ENSG00000157978	AR	Hypercholesterolemia, familial, 4 [7]
PCSK9 *	1p32.3	ENSG00000169174	AD	Hypercholesterolemia, familial, 3 [7]
LIPA	10q23.31	ENSG00000107798	AR	Lysosomal acid lipase deficiency; Cholesteryl ester storage disease [7]
ABHD5	3p21.33	ENSG00000011198	AR	Neutral lipid storage disease with ichthyosis; Chanarin-Dorfman syndrome [7]
PNPLA2	11p15.5	ENSG00000177666	AR	Neutral lipid storage disease with myopathy [8]
LPL	8p21.3	ENSG00000175445	AD	Combined hyperlipidemia, familial [8]
			AR	Lipoprotein lipase deficiency [8]
APOE	19q13.32	ENSG00000130203	AD, AR	Hyperlipoproteinemia, type III [8]
APOC2	19q13.32	ENSG00000234906	AR	Hyperlipoproteinemia, type Ib [8]
LMF1	16p13.3	ENSG00000103227	AR	Lipase deficiency, combined [8]

GPIHBP1	8q24.3	ENSG00000182851	AR	Hyperlipoproteinemia, type 1D [8]
CELA2A	1p36.21	ENSG00000142615	AD	Abdominal obesity-metabolic syndrome 4 [8]
ABCA1	9q31.1	ENSG00000165029	AR	HDL deficiency, familial, 1 [8]
APOA1	11q23.3	ENSG00000118137	AD	Hypoalphalipoproteinemia, primary, 2, with or without corneal clouding [8]
LCAT	16q22.1	ENSG00000213398	AR	Lecithin-cholesterol acyltransferase deficiency; Norum disease [8]
CETP	16q13	ENSG00000087237	AD	Hyperalphalipoproteinemia [8]
LIPC	15q21.3	ENSG00000166035	AR	Hepatic lipase deficiency [8]
SCARB1	12q24.31	ENSG00000073060		High density lipoprotein cholesterol level QTL6 [8]
MTTP *	4q23	ENSG00000138823	AR	Abetalipoproteinemia [9]
APOA5	11q23.3	ENSG00000110243	AD	Hypertriglyceridemia, susceptibility to [10]
GPD1	12q13.12	ENSG00000167588	AR	Hypertriglyceridemia, transient infantile [10]
PPARG	3p25.2	ENSG00000132170	AD	Lipodystrophy, familial partial, type 3 [11]
AGPAT2	9q34.3	ENSG00000169692	AR	Lipodystrophy, congenital generalized, type 1 [11]
BSCL2	11q12.3	ENSG00000168000	AR	Lipodystrophy, congenital generalized, type 2 [11]
CAV1	7q31.2	ENSG00000105974	AR	Lipodystrophy, congenital generalized, type 3 [11]
CAVIN1	17q21.2	ENSG00000177469	AR	Lipodystrophy, congenital generalized, type 4 [11]
LMNA	1q22	ENSG00000160789	AD	Lipodystrophy, familial partial, type 2 [11]
PLIN1	15q26.1	ENSG00000166819	AD	Lipodystrophy, familial partial, type 4 [11]
CIDEC	3p25.3	ENSG00000187288	AR	Lipodystrophy, familial partial, type 5 [11]
LIPE	19q13.2	ENSG00000079435	AR	Lipodystrophy, familial partial, type 6 [11]
**Cholestatic diseases**				
ABCB4	7q21.12	ENSG00000005471	AD, AR	Cholestasis, intrahepatic, of pregnancy, 3 [12]
			AR	Cholestasis, progressive familial intrahepatic 3 [12]
ATP8B1	18q21.31	ENSG00000081923	AR	Cholestasis, progressive familial intrahepatic 1 [12]
			AD	Cholestasis, intrahepatic, of pregnancy, 1
ABCB11	2q31.1	ENSG00000073734	AR	Cholestasis, progressive familial intrahepatic 2 [12]

			AR	Cholestasis, benign recurrent intrahepatic, 2 [12]
TJP2	9q21.11	ENSG00000119139	AR	Cholestasis, progressive familial intrahepatic 4 [12]
NR1H4	12q23.1	ENSG00000012504	AR	Cholestasis, progressive familial intrahepatic, 5 [12]
**Storage Diseases**				
GBA	1q22	ENSG00000177628	AR	Gaucher disease, perinatal lethal; type I; type II; type III; type IIIC [13]
SMPD1	11p15.4	ENSG00000166311	AR	Niemann-Pick disease, type A and B [13]
NPC1	18q11.2	ENSG00000141458	AR	Niemann-Pick disease, type C1 and type D [13]
NPC2	14q24.3	ENSG00000119655	AR	Niemann-pick disease, type C2 [13]
G6PC	17q21.31	ENSG00000131482	AR	Glycogen storage disease Ia [13]
SLC37A4	11q23.3	ENSG00000137700	AR	Glycogen storage disease Ib [13]
GAA	17q25.3	ENSG00000171298	AR	Glycogen storage disease II (Pompe disease) [13]
AGL	1p21.2	ENSG00000162688	AR	Glycogen storage disease IIIb [13]
GBE1	3p12.2	ENSG00000114480	AR	Glycogen storage disease IV [13]
PYGL	14q22.1	ENSG00000100504	AR	Glycogen storage disease VI [13]
PHKA2	Xp22.13	ENSG00000044446	XLR	Glycogen storage disease IXa1 [13]
PHKG2	16p11.2	ENSG00000156873	AR	Glycogen storage disease IXc [13]
PHKB	16q12.1	ENSG00000102893	AR	Glycogen storage disease IXb [13]
SLC2A2	3q26.2	ENSG00000163581	AR	Fanconi-Bickel syndrome [13]
MAN2B1	19p13.13	ENSG00000104774	AR	Mannosidosis, alpha-, types I and II [13]
**Specific Hereditary Liver Diseases**				
ATP7B	13q14.3	ENSG00000123191	AR	Wilson disease [14]
SERPINA1	14q32.13	ENSG00000197249	AR	Alpha-1-antitrypsin deficiency [15]
ALDOB	9q31.1	ENSG00000136872	AR	Fructose intolerance, hereditary [16]
ALMS1	2p13.1	ENSG00000116127	AR	Alstrom syndrome [17]
ASL	7q11.21	ENSG00000126522	AR	Argininosuccinic aciduria [18]
FAH	15q25.1	ENSG00000103876	AR	Tyrosinemia, type I [19]
SLC25A13	7q21.3	ENSG00000004864	AR	Citrullinemia, adult-onset and neonatal-onset, type II [20]

DGUOK	2p13.1	ENSG00000114956	AR	Portal hypertension, noncirrhotic; Mitochondrial DNA depletion syndrome 3 [21]
JAG1	20p12.2	ENSG00000101384	AD	Alagille syndrome 1 [22]
**Susceptibility to liver diseases**				
PNPLA3	22q13	ENSG00000100344	Mu	Fatty liver disease, nonalcoholic, susceptibility to, 1 [23]
MBOAT7 *	19q13.42	ENSG00000125505	AR	Mental retardation, autosomal recessive 57 [23]
TM6SF2	19p13.11	ENSG00000213996		// [23]
GCKR	2p23.3	ENSG00000084734		// [23]
HSD17B13	4q22.1	ENSG00000170509		// [23]
PCSK7		ENSG00000160613		// [24]
ATG7	3p25.3	ENSG00000197548		// [25]
IRF3	19q13.33	ENSG00000126456	AD	Encephalopathy, acute, infection-induced (herpes-specific), susceptibility to, 7 [26]
NMBR	6q24.1	ENSG00000135577		// [27]
RTEL1	20q13.33	ENSG00000258366	AD	Pulmonary fibrosis and/or bone marrow failure, telomere-related, 3 [28]
TERC	3q26.2	ENSG00000270141	AD	Pulmonary fibrosis, idiopathic, susceptibility to; Aplastic anemia [29]
TERT *	5p15.33	ENSG00000164362	AD	Pulmonary fibrosis and/or bone marrow failure, telomere-related, 1 [29]

Supplementary Table 1. Genes (n=82) included in the targeted panel sequencing (TS) design divided into six categories: iron overload, lipid metabolism, cholestatic diseases, storage diseases, specific hereditary liver diseases and gene associated with susceptibility to common liver diseases. For each gene, the coding exons with 25 flanking base pairs (bp) were considered; for genes marked by *, 5'-/3'-untranslated regions (UTRs) and promoter regions were also included.
Abbreviations: AD: autosomal dominant; AR: autosomal recessive; Mu: multifactorial

Supplementary references

[1] Girelli D. Blood. 2022 doi: 10.1182/blood.2021011338.
[2] Celma Nos F. Int J Mol Sci. 2021 doi: 10.3390/ijms22115451.
[3] Romero-Cortadellas L. Int J Mol Sci. 2022 doi: 10.3390/ijms23084406
[4] Corradini E. J Hepatol. 2021 doi: 10.1016/j.jhep.2021.03.014.
[5] Camaschella C. Nat Genet. 2009. doi: 10.1038/ng0409-386.
[6] Burnett JR. GeneReview 2021
[7] Brautbar A. Curr Atheroscler Rep. 2015. doi: 10.1007/s11883-015-0491-z.
[8] Taghizadeh E. Biochemical Genetics 2022 doi.org/10.1007/s10528-021-10130-2
[9] Takahashi M. J Atheroscler Thromb. 2021. doi: 10.5551/jat.RV17056.
[10] Musambil M. Curr Diabetes Rev. 2020 doi: 10.2174/1573399815666190502164131.
[11] Lightbourne M. Endocrinol Metab Clin North Am. 2017 doi:10.1016/j.ecl.2017.01.012.

[12] Amirneni S. World J Gastroenterol 2020 doi: 10.3748/wjg.v26.i47.7470
[13] Platt FM. Nat Rev Dis Primers 2018. doi: 10.1038/s41572-018-0025-4.
[14] Weiss KH. GeneReviews 2016
[15] Stoller JK. GeneReviews 2020
[16] Gaughan . GeneReview 2021
[17] Paisey RB. GeneReview 2019
[18] Nagamani SCS. GeneReview 2019
[19] King L. GeneReview 2017
[20] Saheki T. GeneReview 2017
[21] El-Hattab A. GeneReview 2016
[22] Spinner NB. GeneReview 2019
[23] Bianco C. J Hepatol. 2021 doi: 10.1016/j.jhep.2020.11.024.
[24] Dongiovanni P. J Lipid Res. 2019 doi: 10.1194/jlr.P090449
[25] Baselli GA. J Hepatol. 2022 doi: 10.1016/j.jhep.2022.03.031
[26] Patel SJ. Sci Transl Med 2022.doi: 10.1126/scitranslmed.abh3831.
[27] Rametta R. Am J Hematol 2020. doi: 10.1002/ajh.25679.
[28] Chiu V. Hepatology. 2019. doi: 10.1002/hep.30557.
[29] Donati B. Int J Mol Sci 2016. doi: 10.3390/ijms17030383.
